# Allylic cross-coupling using aromatic aldehydes as α-alkoxyalkyl anions

**DOI:** 10.3762/bjoc.16.21

**Published:** 2020-02-07

**Authors:** Akihiro Yuasa, Kazunori Nagao, Hirohisa Ohmiya

**Affiliations:** 1 Division of Pharmaceutical Sciences, Graduate School of Medical Sciences, Kanazawa University, Kakuma-machi, Kanazawa 920-1192, Japan

**Keywords:** aldehyde, copper, copper catalysis, cross-coupling, palladium, synthetic method

## Abstract

The allylic cross-coupling using aromatic aldehydes as α-alkoxyalkyl anions is described. The synergistic palladium/copper-catalyzed reaction of aromatic aldehydes, allylic carbonates, and a silylboronate produces the corresponding homoallylic alcohol derivatives. This process involves the catalytic formation of a nucleophilic α-silyloxybenzylcopper(I) species and the subsequent palladium-catalyzed allylic substitution.

## Introduction

**Scheme 1:**
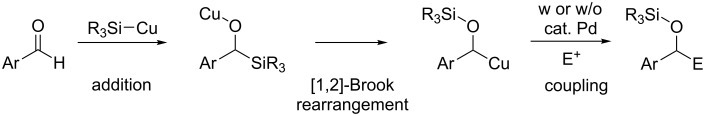
Our strategy.

α-Alkoxy-substituted carbanions (α-alkoxyalkyl anions) are useful C(sp^3^) nucleophiles for the construction of alcohol units found in a majority of pharmaceuticals, agrochemicals and bioactive natural products. Generally, α-alkoxyalkyl anions are presynthesized as stoichiometric organometallic reagents such as organolithium, organozinc, organocuprate, organostannane, organosilane and organoboron compounds [1–6]. Alternatively, we showed that easily available aromatic aldehydes can be used as α-alkoxyalkyl anions for catalytic carbon–carbon bond formations [7–9]. For example, a nucleophilic α-silyloxybenzylcopper(I) species can be generated catalytically from aromatic aldehydes through the 1,2-addition of a silylcopper(I) species followed by [1,2]-Brook rearrangement and then successfully trapped with aryl bromides under palladium catalysis (Scheme 1). This system was extended to an asymmetric version using the chiral α-silyloxybenzylcopper(I) species having a chiral NHC ligand. In the asymmetric system, one example of allylic carbonate was used as the carbon electrophile [8,10–11]. This paper describes in full detail the racemic system using allylic carbonates. The allylic cross-coupling of aromatic aldehydes and allylic carbonates with a silylboronate by the merging of a copper–*N*-heterocyclic carbene catalyst and a palladium–bisphosphine catalyst produced homoallylic alcohol derivatives [12–14].

## Results and Discussion

**Scheme 2:**
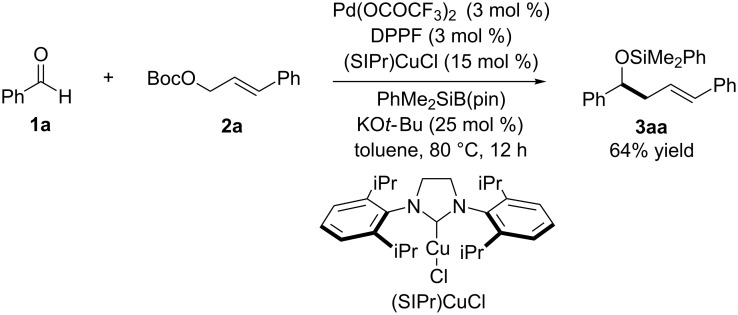
Allylic cross-coupling using aldehydes as α-alkoxyalkyl anions.

Specifically, the three-component allylic cross-coupling reaction of benzaldehyde (**1a**, 0.4 mmol), *tert*-butyl cinnamyl carbonate (**2a**, 0.2 mmol) and (dimethylphenylsilyl)boronic acid pinacol ester [PhMe_2_SiB(pin)] (0.4 mmol) occurred in the presence of catalytic amounts of Pd(OCOCF_3_)_2_ (3 mol %), DPPF (3 mol %), (SIPr)CuCl (15 mol %) and KO*t-*Bu (25 mol %) in toluene at 80 °C to afford homoallylic alcohol derivative **3aa** in 64% yield (based on **2a**) (Scheme 2). The reaction yielded small amounts of side products such as cinnamylsilane and benzyl silyl ether, which are derived from the Pd-catalyzed allylic silylation of **2a** and the Cu-catalyzed silylation of **1a** and the subsequent [1,2]-Brook rearrangement, respectively. In this coupling reaction, (SIPr)CuCl was a slightly better copper complex than (IPr)CuCl (62%), (SIMes)CuCl (60%) and (IMes)CuCl (53%) in terms of the chemical yield. Notably, the allylic cross-coupling reaction did not occur at all without Pd(OCOCF_3_)_2_–DPPF or (SIPr)CuCl, and thus the palladium and copper catalysts cooperatively acted in the allylic cross-coupling.

**Scheme 3:**
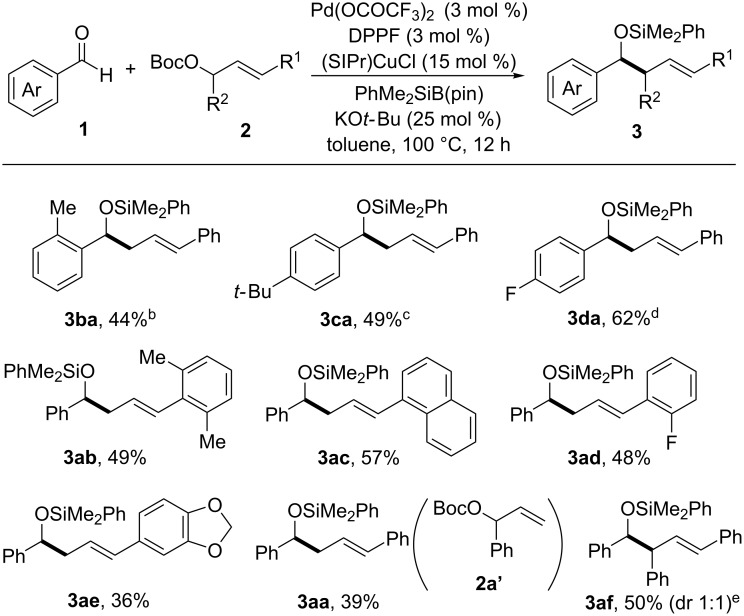
Substrate scope and reaction conditions. a) reactions were carried out with **1** (0.4 mmol), **2** (0.2 mmol), PhMe_2_SiBpin (0.4 mmol), Pd(OCOCF_3_)_2_ (3 mol %), DPPF (3 mol %), (SIPr)CuCl (15 mol %), KO*t-*Bu (25 mol %) in toluene (2 mL) at 100 °C for 12 h. b) Pd(OCOCF_3_)_2_/DPPF (3 mol %), (SIPr)CuCl (25 mol %) and KO*t-*Bu (35 mol %) were used and the reaction temperature was decreased to 80 °C. c) Pd(OCOCF_3_)_2_/DPPF (3 mol %), (SIPr)CuCl (25 mol %) and KO*t-*Bu (35 mol %) were used. d) The reaction temperature was decreased to 80 °C. e) Pd(OCOCF_3_)_2_/DPPF (5 mol %), (SIPr)CuCl (25 mol %) and KO*t-*Bu (35 mol %) were used.

Scheme 3 shows the substrate range of aromatic aldehydes **1** and allylic carbonates **2**. Methyl, *tert*-butyl and fluoro substituents were tolerated at the *ortho*- or *para*-positions of the aromatic aldehyde (**3ba**–**da**). 2,6-Dimethylphenyl- or 1-naphthyl moieties as the γ-substituent of the primary allylic carbonate were tolerated in the reaction (**3ab** and **3ac**). Cinnamyl carbonates having a fluoro or acetal substituent were also suitable coupling partners (**3ad** and **3ae**).

The synergistic palladium/copper catalysis was used for the reaction of a secondary allylic carbonate. For example, the allylic cross-coupling of **2a’**, a constitutional isomer of **2a**, with benzaldehyde (**1a**) afforded the linear allylation product **3aa** with complete regioselectivity. The symmetric secondary allylic carbonate was converted to the corresponding homoallylic alcohol derivative in 50% yield (**3af**).

**Scheme 4:**
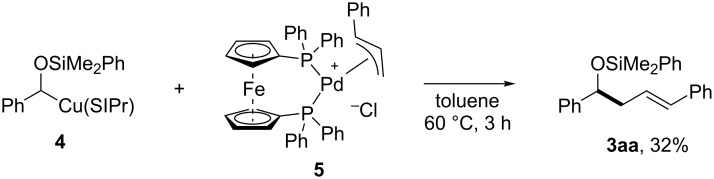
Stoichiometric reaction.

To gain understanding into the mechanism of this synergistic palladium/copper-catalyzed allylic cross-coupling, a stoichiometric experiment was conducted (Scheme 4). The reaction of the SIPr-ligated α-silyloxybenzylcopper **4** with the cinnamyl–palladium complex **5**, which was prepared in situ from [(cinnamyl)PdCl]_2_ and DPPF, gave the corresponding homoallylic alcohol derivative **3aa**.

**Scheme 5:**
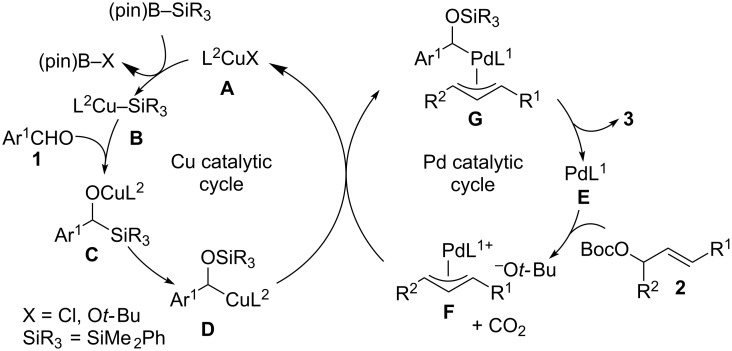
Possible pathway.

Based on previous reports and the outcome obtained by the stoichiometric experiment in Scheme 4, a possible reaction pathway involving a cooperative action of palladium and copper catalysis can be proposed as illustrated in Scheme 5. The reaction of SIPr-ligated CuCl (**A**), KO*t-*Bu and a silylboronate produces a silylcopper(I) species **B**. The 1,2-addition of silylcopper(I) **B** to the aromatic aldehyde **1** [15–19] and the subsequent [1,2]-Brook rearrangement from the obtained α-silyl-substituted copper(I) alkoxide **C** forms the key intermediate, an α-silyloxybenzylcopper(I) species **D**. The transmetallation between **D** and an allylpalladium(II) species **F** that is generated through the oxidative addition of an allylic carbonate **2** across a palladium(0)–DPPF complex **E**, followed by reductive elimination from **G** produces the homoallylic alcohol **3** and then regenerate **A** and **E** for the next catalytic cycle [20–23].

## Conclusion

In summary, we developed an umpolung strategy for catalytically formed α-alkoxyalkyl anions from aromatic aldehydes for the use in allylic cross-coupling reactions. The synergistic palladium/copper-catalyzed reaction of aromatic aldehydes, allylic carbonates, and a silylboronate delivered the homoallylic alcohol derivatives. This process involves the catalytic formation of a nucleophilic α-silyloxybenzylcopper(I) species and the subsequent palladium-catalyzed allylic substitution.

## Experimental

SIPrCuCl (14.7 mg, 0.03 mmol), and KO*t*-Bu (4.5 mg, 0.04 mmol) were placed in a vial containing a magnetic stirring bar. The vial was sealed with a Teflon^®^-coated silicon rubber septum, and then the vial was evacuated and filled with nitrogen. Toluene (0.6 mL) was added to the vial, and then the mixture was stirred at 25 °C for 10 min. Next, PhMe_2_SiB(pin) (104.9 mg, 0.4 mmol) and benzaldehyde (**1a**, 42.4 mg, 0.4 mmol) were added, and the mixture (mixture A) was stirred at 25 °C for 10 min. Meanwhile, Pd(OCOCF_3_)_2_ (2.0 mg, 0.006 mmol) and DPPF (3.3 mg, 0.006 mmol) were placed in another vial. This vial was sealed with a Teflon^®^-coated silicon rubber septum and then evacuated and filled with nitrogen. After toluene (0.8 mL) was added to the vial, the mixture was stirred at 25 °C for 10 min. Next, KO*t-*Bu (1.1 mg, 0.01 mmol) and allylic carbonate **2a** (46.9 mg, 0.2 mmol) were added to the vial, and the mixture (mixture B) was stirred at 25 °C for 10 min. Finally, the palladium solution (mixture B) was transferred to the vial (mixture A) containing the copper complex with toluene (0.6 mL). After 12 h stirring at 80 °C, the reaction mixture was diluted with diethyl ether (1 mL). The reaction mixture was filtered through a short plug of aluminum oxide (1 g) with diethyl ether as an eluent. After volatiles were removed under reduced pressure, GPC (EtOAc) followed by flash chromatography on silica gel (0–1% EtOAc/hexane) gave product **3aa** in 64% isolated yield (45.7 mg, 0.13 mmol).

## Supporting Information

File 1 Experimental procedures, spectroscopic and analytical data, and copies of NMR spectra for newly synthesized compounds.
