# Supplementary material for: Allylic cross-coupling using aromatic aldehydes as α-alkoxyalkyl anions
Source: Beilstein J Org Chem. 2020 Feb 7;16:185–9. doi: 10.3762/bjoc.16.21 (PMC7034225; doi:10.3762/bjoc.16.21)
Supplement: File 1 — Experimental procedures, spectroscopic and analytical data, and copies of NMR spectra for newly synthesized compounds. [file Beilstein_J_Org_Chem-16-185-s001.pdf]

BEILSTEIN JOURNAL OF ORGANIC CHEMISTRY

Supporting Information

for

Allylic cross-coupling using aromatic aldehydes as α-alkoxyalkyl anions

Akihiro Yuasa, Kazunori Nagao and Hirohisa Ohmiya

Beilstein J. Org. Chem. **2020,** *16,* 185–189. doi:10.3762/bjoc.16.21

Experimental procedures, spectroscopic and analytical data, and copies of NMR spectra for newly synthesized compounds

License and Terms: This is a supporting information file under the terms of the Creative Commons Attribution License (https://creativecommons.org/licenses/by/4.0). Please note that the reuse, redistribution and reproduction in particular requires that the authors and source are credited.
The license is subject to the *Beilstein Journal of Organic Chemistry* terms and conditions: (https://www.beilstein-journals.org/bjoc)

Table of Contents

- Instrumentation and chemicals S2
- Characterization data for homoallylalcohols S3
- Procedure for stoichiometric experiment S6
- References S6
- NMR spectra S7

■ **Instrumentation and chemicals** ■

NMR spectra were recorded on a JNM-ECS400, operating at 400 MHz for ^{1}H NMR and 100.5 MHz for ^{13}C NMR, and JNM-ECA600, operating at 600 MHz for ^{1}H NMR and 150.9 MHz for ^{13}C NMR. Chemical shift values for ^{1}H and ^{13}C are referenced to Me_4Si and the residual solvent resonances, respectively. Chemical shifts are reported in δ ppm. Mass spectra were obtained with a JMS-T100TD (DART). TLC analyses were performed on commercial glass plates with a 0.25-mm layer of Merck silica gel $60F_{254}$. Silica gel (Kanto Chemical Co., Silica gel 60 N, spherical, neutral) was used for column chromatography. IR spectra were measured with a Thermo Scientific iD7 ATR Accessory for the Thermo Scientific Nicolet iS5 FT-IR Spectrometer. Melting points were measured on a Yanaco MP-500D apparatus. Gel permeation chromatography (GPC) was performed by LC-908 (Japan Analytical Industry Ltd., two in-line JAIGEL-2H, EtOAc, 3.5 mL/min, UV and RI detectors).

All reactions were carried out under nitrogen or argon atmosphere. Materials were obtained from commercial suppliers or prepared according to standard procedures unless otherwise noted. Toluene was purchased from Wako Pure Chemical Industries, stored under nitrogen, and used as received. CuCl was purchased from Aldrich Chemical Co., stored under nitrogen, and used as received. $PhMe_2SiB(pin)$ was purchased from Wako Pure Chemical Industries, stored under nitrogen, and used as received. KO*t*-Bu was purchased from Tokyo Chemical Industry Co., stored under nitrogen, and used as received. $Pd(TFA)_2$ was purchased from Tokyo Chemical Industry Co., stored under nitrogen, and used as received. DPPF were purchased from Kanto Chemical Co., stored under nitrogen, and used as received. (SIPr)CuCl was prepared according to the literature.[1] Allylic carbonates were prepared by Boc protection of the corresponding allylic alcohols.

■ **Characterization data for homoallylalcohols** ■

(*E*)-[(1,4-Diphenylbut-3-en-1-yl)oxy]dimethylphenylsilane (3aa)

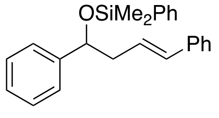

3aa

The product **3aa** was purified by flash chromatography on silica gel (100:0–99:1, hexane/EtOAc) and GPC (EtOAc) (with **2a** in Scheme 2; 45.7 mg, 0.13 mmol, 64% isolated yield, with **2a'** in Scheme 3; 28.0 mg, 0.078 mmol, 39% isolated yield). The compound is contaminated with inseparable impurities. Colorless Oil. **IR** (neat) 1068, 1116, 1251, 1427 cm^{-1}. **^{1}H NMR** (600 MHz, $CDCl_3$) δ 0.25 (s, 3H), 0.29 (s, 3H), 2.53 (m, 1H), 2,63 (m, 1H), 4.74 (dd, *J* = 7.8, 5.4 Hz, 1H), 6.12 (dt, *J* = 15.0, 7.2 Hz, 1H), 6.35 (d, *J* = 15.6 Hz, 1H), 7.18–7.37 (m, 13H), 7.50–7.52 (m, 2H). **^{13}C NMR** (150 MHz, $CDCl_3$) δ –1.4, −0.9, 44.2, 75.4, 125.9, 126.0, 126.9, 127.0, 127.1, 127.7, 128.1, 128.4, 129.5, 132.1, 133.6, 137.6, 137.9, 144.6. **HRMS–DART** (*m/z*): $[M–H]^+$ calcd for $C_{24}H_{25}OSi$, 357.1675; found, 357.1675

(*E*)-Dimethylphenyl{[4-phenyl-1-(*o*-tolyl)but-3-en-1-yl]oxy}silane (3ba)

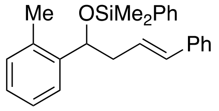

3ba

The product **3ba** was purified by flash chromatography on silica gel (100:0–99:1, hexane/EtOAc) and GPC (EtOAc) (Scheme 3; 32.5 mg, 0.09 mmol, 44% isolated yield). Colorless Oil. The spectrum data of product **3ba** was consistent with the literature.[2]

(*E*)-{[1-(4-*tert*-Butylphenyl)-4-phenylbut-3-en-1-yl]oxy}dimethylphenylsilane (3ca)

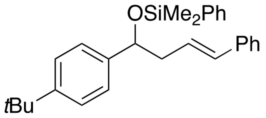

3ca

The product **3ca** was purified by flash chromatography on silica gel (100:0–99:1, hexane/EtOAc) and GPC (EtOAc) (Table 1; 40.5 mg, 0.10 mmol, 49% isolated yield). Colorless Oil. **IR** (neat) 679, 786, 829, 1080, 1117, 2961 cm^{-1}. **^{1}H NMR** (600 MHz, $CDCl_3$) δ 0.24 (s, 3H), 0.29 (s, 3H), 1.32 (s, 9H), 2.53 (m, 1H), 2,63 (m, 1H), 4.72 (dd, *J* = 7.8, 4.8 Hz, 1H), 6.14 (dt, *J* = 15.6, 7.2 Hz, 1H), 6.35 (d, *J* = 15.6 Hz, 1H), 7.18–7.22 (m, 3H), 7.26–7.32 (m, 8H), 7.35 (m, 1H), 7.49–7.51 (m, 2H). **^{13}C NMR** (150.9 MHz, $CDCl_3$) δ –1.4, –0.9, 31.4, 34.5, 44.2, 75.3, 124.9, 125.6, 126.0, 126.9, 127.4, 127.7, 128.4, 129.4, 132.0, 133.6, 137.7, 138.0, 141.5, 149.9. **HRMS–DART** (*m/z*): $[M+NH_4]^+$ calcd for $C_{28}H_{38}NOSi$, 432.2723; found, 432.2727.

(*E*)-{[1-(4-Fluorophenyl)-4-phenylbut-3-en-1-yl]oxy}dimethylphenylsilane (3da)

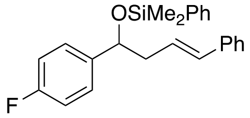

3da

The product **3da** was purified by flash chromatography on silica gel (100:0–99:1, hexane/EtOAc) and GPC (EtOAc) (Scheme 3; 46.8 mg, 0.12 mmol, 62% isolated yield). Colorless Oil. **IR** (neat) 669, 742, 787, 829, 1081, 1117, 1227, 1222, 1508 cm^{-1}. ^{1}**H NMR** (600 MHz, $CDCl_3$) δ 0.25 (s, 3H), 0.30 (s, 3H), 2.50 (m, 1H), 2,61 (m, 1H), 4.71 (t, *J* = 7.2 Hz, 1H), 6.08 (dt, *J* = 16.2, 7.2 Hz, 1H), 6.38 (d, *J* = 16.2 Hz, 1H), 6.98–6.99 (m, 2H), 7.20 (m, 1H), 7.23–7.31 (m, 8H), 7.37 (m, 1H), 7.49–7.50 (m, 2H). ^{13}**C NMR** (150.9 MHz, $CDCl_3$) δ –1.4, –1.0, 44.2, 74.8, 114.8, 115.0, 126.5, 127.0, 127.5 (d, *J* = 7.2 Hz), 127.7, 128.4, 129.6, 132.4, 133.5, 137.6 (d, *J* = 21.5 Hz), 140.3 (d, *J* = 2.9 Hz), 161.9 (d, *J* = 245.6 Hz). **HRMS–DART** (*m/z*): $[M+NH_4]^+$ calcd for $C_{24}H_{29}FNOSi$, 394.2002; found, 394.2004.

(*E*)-{[4-(2,6-Dimethylphenyl)-1-phenylbut-3-en-1-yl]oxy}dimethylphenylsilane (3ab)

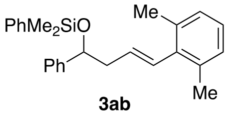

3ab

The product **3ab** was purified by flash chromatography on silica gel (100:0–99:1, hexane/EtOAc) and GPC (EtOAc) (Scheme 3; 37.8 mg, 0.10 mmol, 49% isolated yield). Colorless Oil. **IR** (neat) 669, 768, 785, 829, 1082, 1116, 1252 cm^{-1}. ^{1}**H NMR** (600 MHz, $CDCl_3$) δ 0.25 (s, 3H), 0.29 (s, 3H), 2.14 (s, 6H) 2.63 (m, 1H), 2,71 (m, 1H), 4.79 (t, *J* = 6.0 Hz, 1H), 5.52 (dt, *J* = 16.2, 7.2 Hz, 1H), 6.22 (d, *J* = 16.2 Hz, 1H), 6.96–7.01 (m, 3H), 7.21 (m, 1H), 7.25–7.32 (m, 6H), 7.36 (m, 1H), 7.51–7.52 (m, 2H). ^{13}**C NMR** (150.9 MHz, $CDCl_3$) δ –1.4, –0.9, 20.9, 44.4, 75.2, 126.1, 126.2, 127.1, 127.5, 127.7, 128.1, 129.5, 129.9, 131.1, 133.5, 135.9, 137.4, 137.9, 144.2. **HRMS–DART** (*m/z*): $[M+NH_4]^+$ calcd for $C_{26}H_{34}NOSi$, 404.2410; found, 404.2407.

(*E*)-Dimethyl[(4-(naphthalen-1-yl)-1-phenylbut-3-en-1-yl)oxy]phenylsilane (3ac)

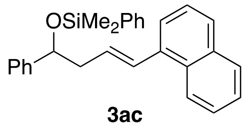

3ac

The product **3ac** was purified by flash chromatography on silica gel (100:0–99:1, hexane/EtOAc) and GPC (EtOAc) (Scheme 3; 46.2 mg, 0.11 mmol, 57% isolated yield). Colorless Oil. **IR** (neat) 669, 740, 786, 828, 1065, 1116, 1251 cm^{-1}. ^{1}**H NMR** (600 MHz, $CDCl_3$) δ 0.27 (s, 3H), 0.31 (s, 3H), 2.67 (m, 1H), 2,76 (m, 1H), 4.83 (t, *J* = 6.6 Hz, 1H), 6.11 (dt, *J* = 15.6, 6.6 Hz, 1H), 7.02 (d, *J* = 15.6 Hz, 1H), 7.26–7.47 (m, 13H), 7.53 (m, 1H), 7.02 (d, *J* = 7.8 Hz, 1H), 7.82 (m, 1H), 7.96 (d, *J* = 8.6 Hz, 1H). ^{13}**C NMR** (150.9 MHz, $CDCl_3$) δ –1.4, -0.9, 44.5, 75.3, 123.6, 124.0, 125.6, 125.7, 126.1, 127.2, 127.3, 127.7, 128.1, 128.3 (× 2C), 129.5, 129.6, 130.0, 131.1, 133.5, 133.6, 135.5, 137.9, 144.5. **HRMS–DART** (*m/z*): $[M+NH_4]^+$ calcd for $C_{28}H_{32}NOSi$, 426.2253; found, 426.2254.

(*E*)-[(4-(2-Fluorophenyl)-1-phenylbut-3-en-1-yl)oxy]dimethylphenylsilane (3ad)

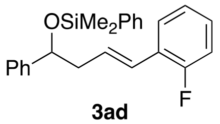

3ad

The product **3ad** was purified by flash chromatography on silica gel (100:0–99:1, hexane/EtOAc) and GPC (EtOAc) (Scheme 3; 36.2 mg, 0.10 mmol, 48% isolated yield). Colorless Oil. **IR** (neat) 669, 752, 785, 828, 1068, 1116, 1486 cm^{-1}. **^{1}H NMR** (600 MHz, $CDCl_3$) δ 0.24 (s, 3H), 0.29 (s, 3H), 2.55 (m, 1H), 2,65 (m, 1H), 4.75 (dd, *J* = 7.2, 5.4 Hz, 1H), 6.20 (dt, *J* = 16.2, 7.2 Hz, 1H), 6.51 (d, *J* = 16.2 Hz, 1H), 6.97–7.06 (m, 2H), 7.13–7.37 (m, 10H), 7.50–7.53 (m, 2H). **^{13}C NMR** (150.9 MHz, $CDCl_3$) δ –1.4, –0.9, 44.6, 75.3, 115.6 (d, *J* = 30 Hz), 123.9 (d, *J* = 3.0 Hz), 124.5 (d, *J* = 3.0 Hz), 125.4 (d, *J* = 12 Hz), 125.9, 127.1 (× 2C), 127.2, 127.7, 128.1, 129.6 (d, *J* = 36 Hz), 129.8, 133.6, 137.9, 144.5, 160.0 (d, *J* = 246 Hz). **HRMS–DART** (*m/z*): $[M+NH_4]^+$ calcd for $C_{24}H_{29}FNOSi$, 394.2002; found, 394.2000.

(*E*)-{[4-(Benzo[*d*][1,3]dioxol-5-yl)-1-phenylbut-3-en-1-yl]oxy}dimethylphenylsilane (3ae)

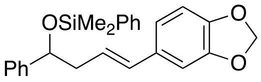

3ae

The product **3ae** was purified by flash chromatography on silica gel (100:0–99:1, hexane/EtOAc) and GPC (EtOAc) (Scheme 3; 46.8 mg, 0.07 mmol, 36% isolated yield). Colorless Oil. **IR** (neat) 410, 700, 785, 829, 1040, 1250, 1489, 1503 cm^{-1}. **^{1}H NMR** (600 MHz, $CDCl_3$) δ 0.24 (s, 3H), 0.28 (s, 3H), 2.49 (m, 1H), 2,59 (m, 1H), 4.71 (dd, *J* = 7.8, 5.4 Hz, 1H), 5.93 (dt, *J* = 15.6, 7.2 Hz, 1H), 5.93 (s, 2H), 6.25 (d, *J* = 15.6 Hz, 1H), 6.67 (d, *J* = 1.2 Hz, 1H), 6.69 (d, *J* = 1.2 Hz, 1H), 6.72 (d, *J* = 7.8 Hz, 1H), 6.79 (s, 1H), 7.22–7.32 (m, 7H), 7.36 (m, 1H), 7.52 (m, 1H). **^{13}C NMR** (150. 9 MHz, $CDCl_3$) δ –1.4, –0.9, 44.1, 75.5, 100.9, 105.4, 108.2, 120.4, 125.2, 125.9, 127.1, 127.7, 128.1, 129.5, 131.7, 132.2, 133.6, 137.9, 144.6, 146.7, 147.9. **HRMS–DART** (*m/z*): $[M+NH_4]^+$ calcd for $C_{25}H_{30}NO_3Si$, 420.1995 ; found, 420.2008

(*E*)-Dimethylphenyl[(1,2,4-triphenylbut-3-en-1-yl)oxy]silane (3af)

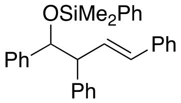

3af

The product **3af** was purified by flash chromatography on silica gel (100:0–99:1, hexane/EtOAc) and GPC (EtOAc) (Scheme 3; 43.2 mg, 0.10 mmol, 50% isolated yield). Diastereomeric ratio is 1:1. Colorless Oil. **IR** (neat) 1067, 1252, 1452, 1494 cm^{-1}. **^{1}H NMR** (400 MHz, $CDCl_3$) δ 0.07 (s, 0.5 × 3H), 0.09 (s, 0.5 × 3H), 0.11 (s, 0.5 × 3H), 0.16 (s, 0.5 × 3H), 3.66–3.72 (m, 0.5 × 1H + 0.5 × 1H), 4.89 (d, *J* = 6.4 Hz, 0.5 × 1H), 4.90 (d, *J* = 7.2 Hz, 0.5 × 1H), 6.14 (d, *J* = 15.6 Hz, 0.5 × 1H), 6.26–6.32 (m, 0.5 × 1H + 0.5 × 1H), 6.64 (dd, *J* = 16.0, 8.8 Hz, 0.5 × 1H), 7. 07-7.41 (m, 0.5 × 20H + 0.5 × 20H). **^{13}C NMR** (100 MHz, $CDCl_3$) δ –1.5 (× 2C), –1.2, –1.1, 58.2, 58.3, 79.4, 79.5, 126.1, 126.2, 126.3 (× 2C),126.8, 127.0 (× 2C), 127.2, 127.6 (× 3C), 127.7, 128.0, 128.1, 128.3, 128.4, 128.7, 129.2,

129.4 (× 2C), 129.9, 130.3, 131.6, 132.0, 133.5 (× 2C), 133.6 (× 2C), 137.5, 137.7 (× 3C), 141.4, 141.8, 142.9, 143.0. **HRMS–DART** (*m/z*): $[M–H]^+$ calcd for $C_{30}H_{29}OSi$, 433.1988; found, 433.1989.

■ Procedure for stoichiometric experiment ■

Scheme 4. (SIPr)CuCl (48.8 mg, 0.08 mmol) and KO*t*-Bu (11.2 mg, 0.08 mmol) were placed in a vial containing a magnetic stirring bar. The vial was sealed with a Teflon®-coated silicon rubber septum, and then evacuated and filled with nitrogen. Toluene (0.6 mL) was added to the vial, and the mixture stirred at 25 °C for 10 min. Next, $PhMe_2SiB(pin)$ (21.0 mg, 0.08 mmol) and benzaldehyde (**1a**, 8.5 mg, 0.08 mmol) were added, and the mixture (mixture A) stirred at 25 °C for 40 min. Meanwhile, $[(cinnamyl)PdCl]_2$ (20.7 mg, 0.04 mmol) and DPPF (44.4 mg, 0.08 mmol) were placed in another vial. This vial was sealed with a Teflon®-coated silicon rubber septum and then evacuated and filled with nitrogen. After toluene (1.2 mL) was added to the vial, the mixture (mixture B) was stirred at 25 °C for 15 min. Finally, the palladium solution (mixture B) was transferred to the vial containing the copper complex (mixture A). After 3 h of stirring at 60 °C, the reaction mixture was diluted with diethyl ether (1 mL) and filtered through a short plug of aluminum oxide (1 g) with diethyl ether as an eluent. After volatiles were removed under reduced pressure, flash chromatography on silica gel (0–1% EtOAc/hexane) and GPC ($CHCl_3$) produdt **3aa** (9.2 mg, 0.026 mmol) was obtained in 32% yield.

■ References■

(1) Santoro, O.; Collado, A.; Slawin, A. M. Z.; Nolan, S. P.; Cazin, C. S. J. *Chem. Commun*. **2013**, *49*, 10483−10485.

(2) Yabushita, K.; Yuasa, A.; Nagao, K.; Ohmiya, H. *J. Am. Chem. Soc.* **2019**, *141*, 113–117.

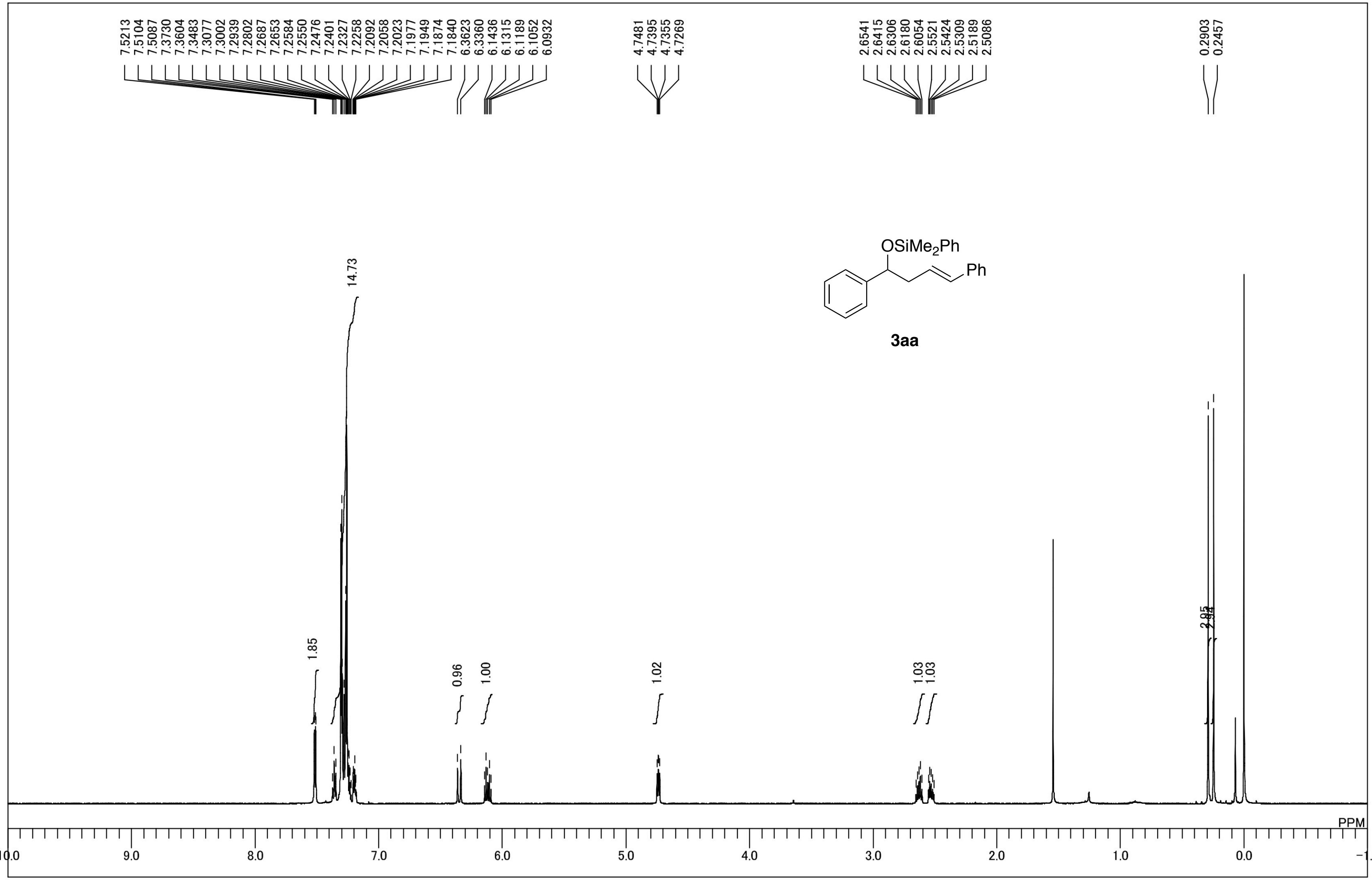

^{1}H NMR spectrum of **3aa**

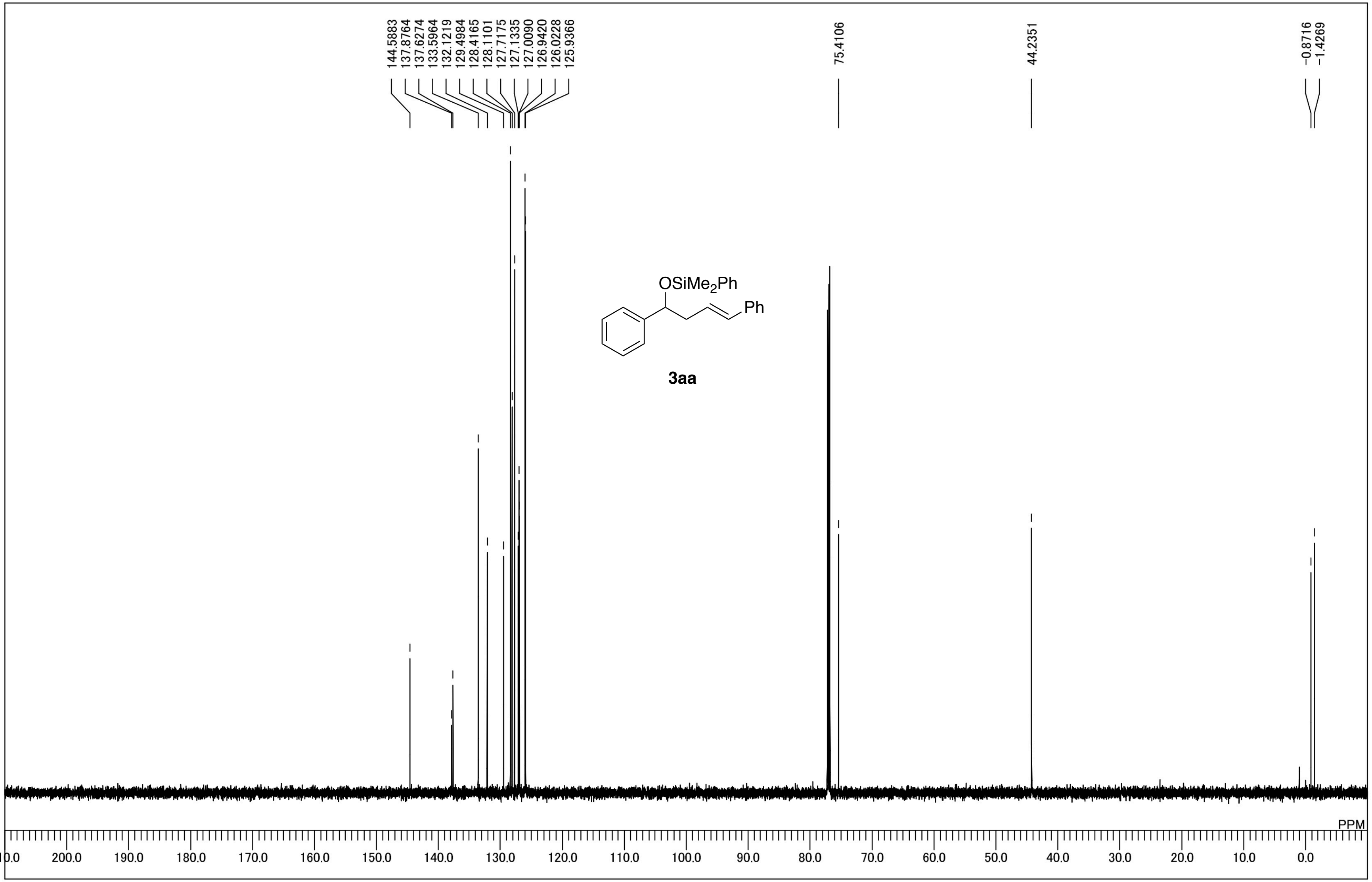

^{13}C NMR spectrum of **3aa**

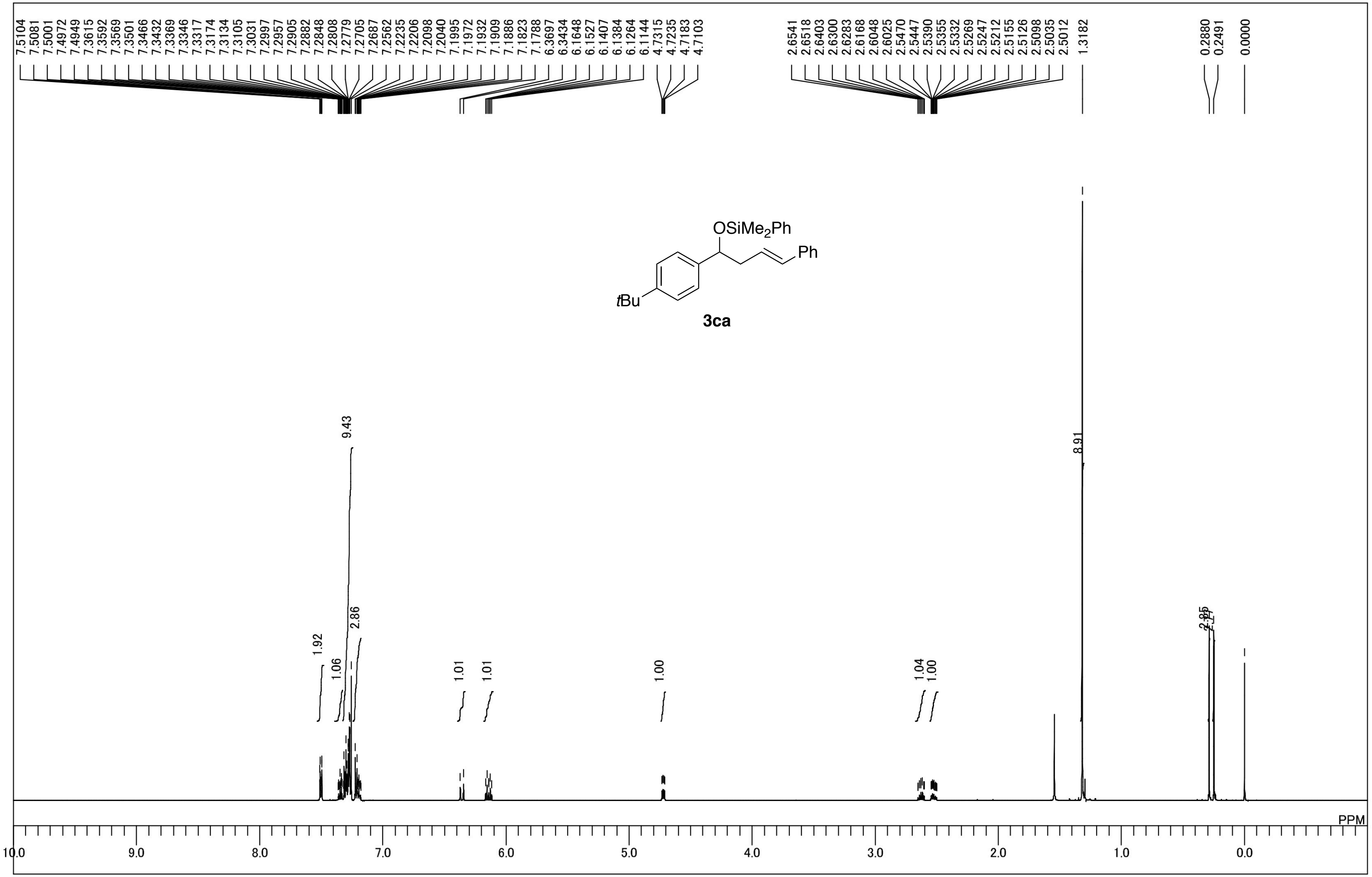

^{1}H NMR spectrum of **3ca**

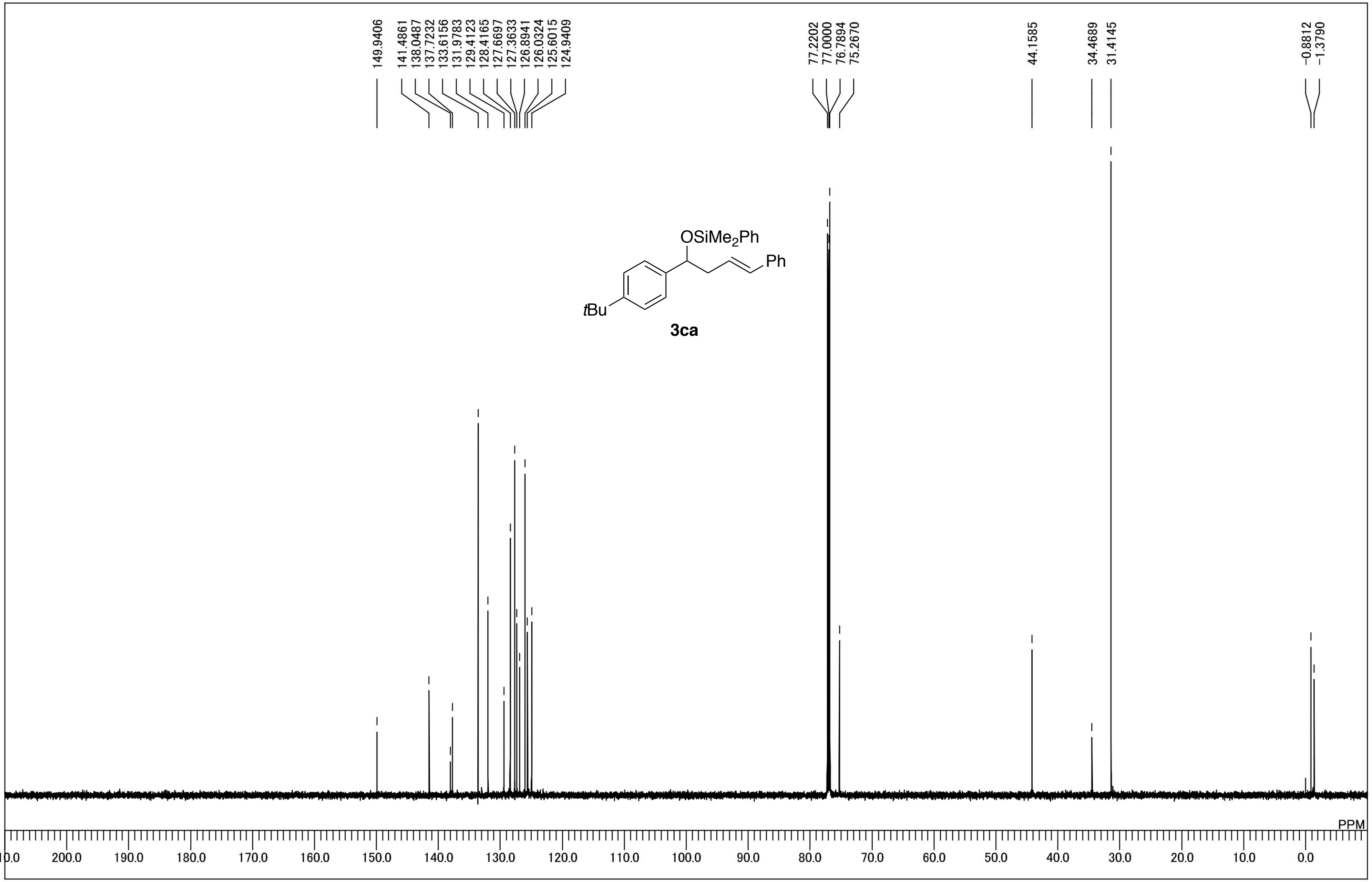

^{13}C NMR spectrum of **3ca**

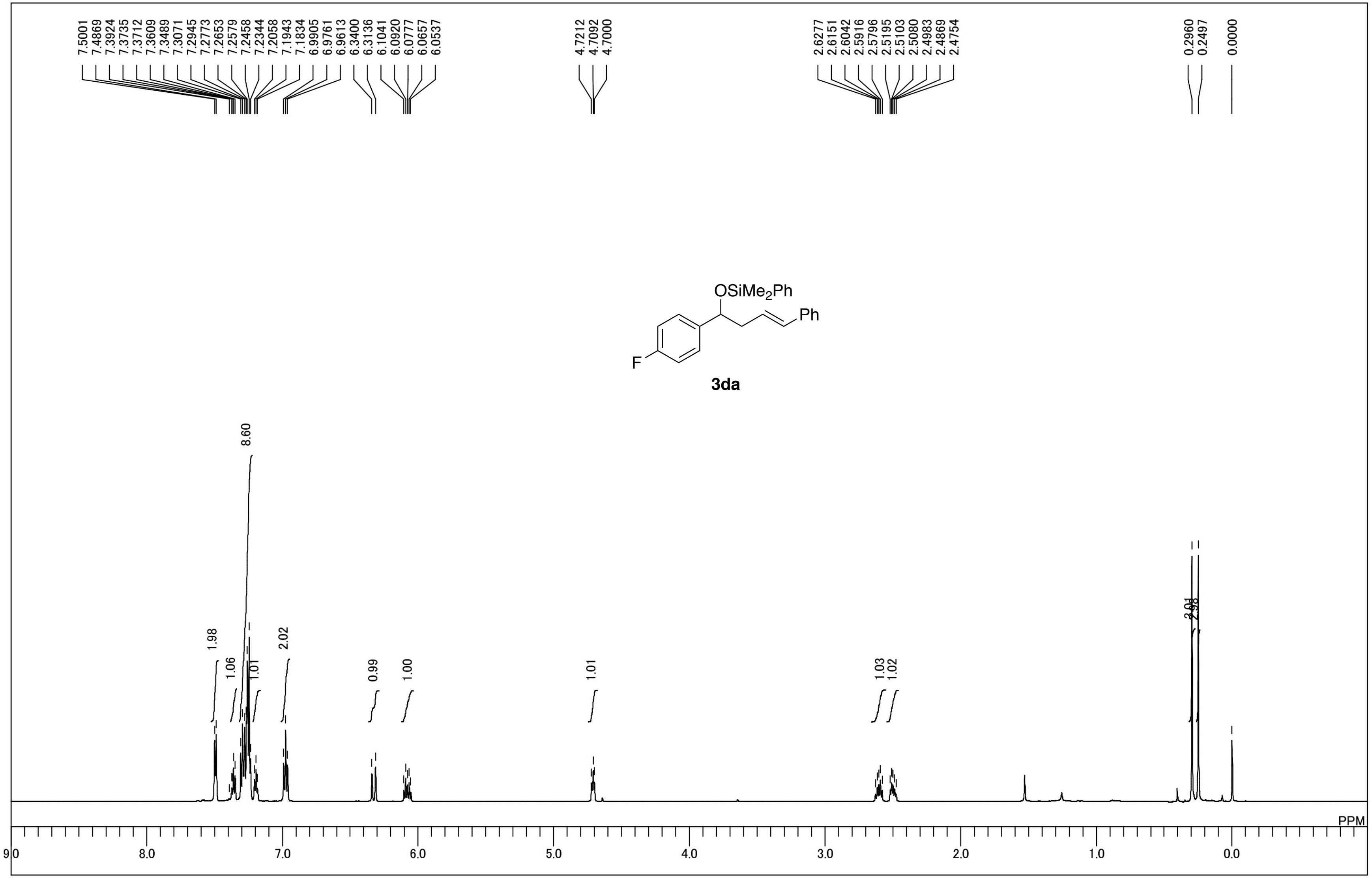

^{1}H NMR spectrum of **3da**

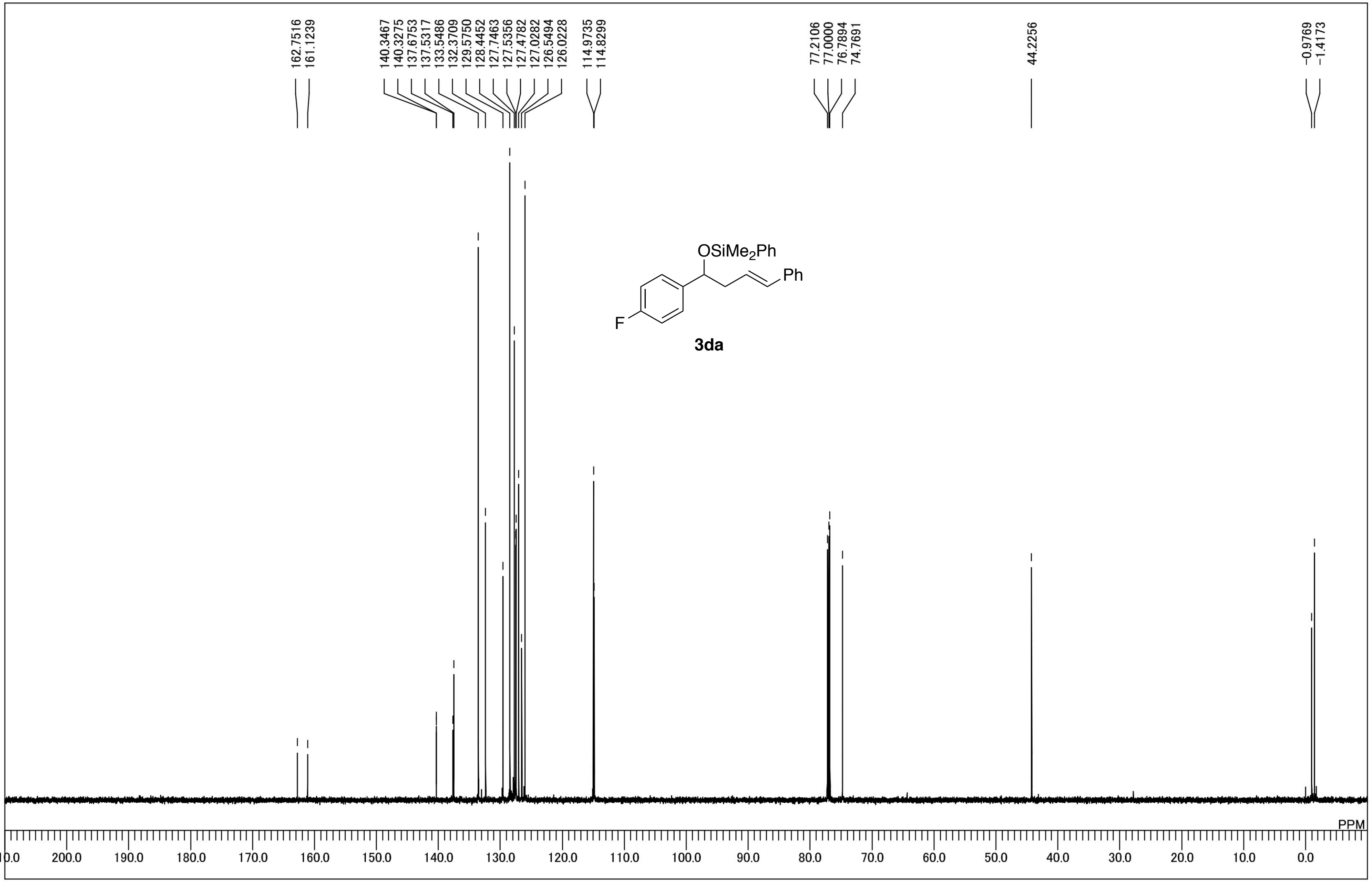

^{13}C NMR spectrum of **3da**

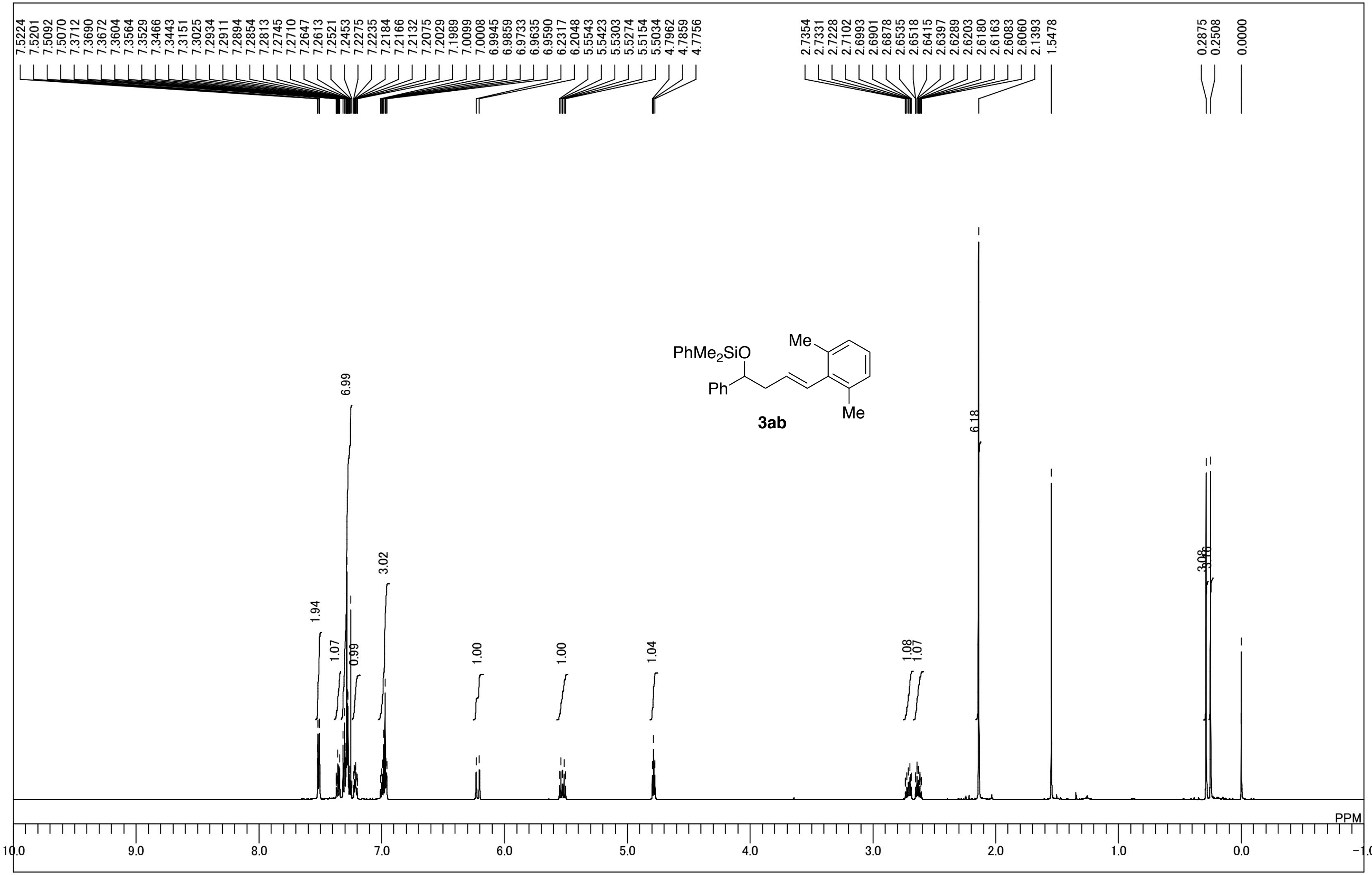

^{1}H NMR spectrum of **3ab**

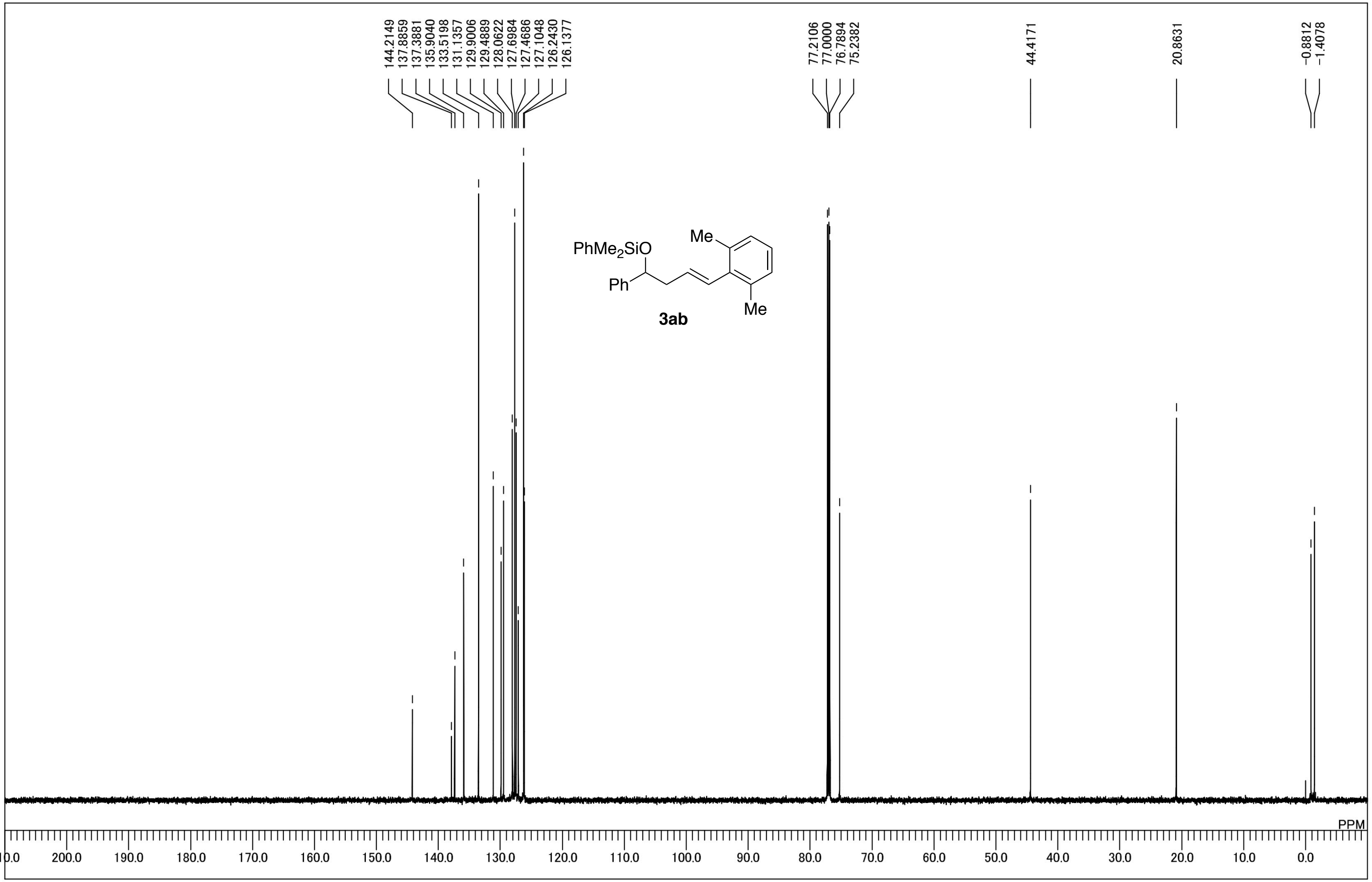

^{13}C NMR spectrum of **3ab**

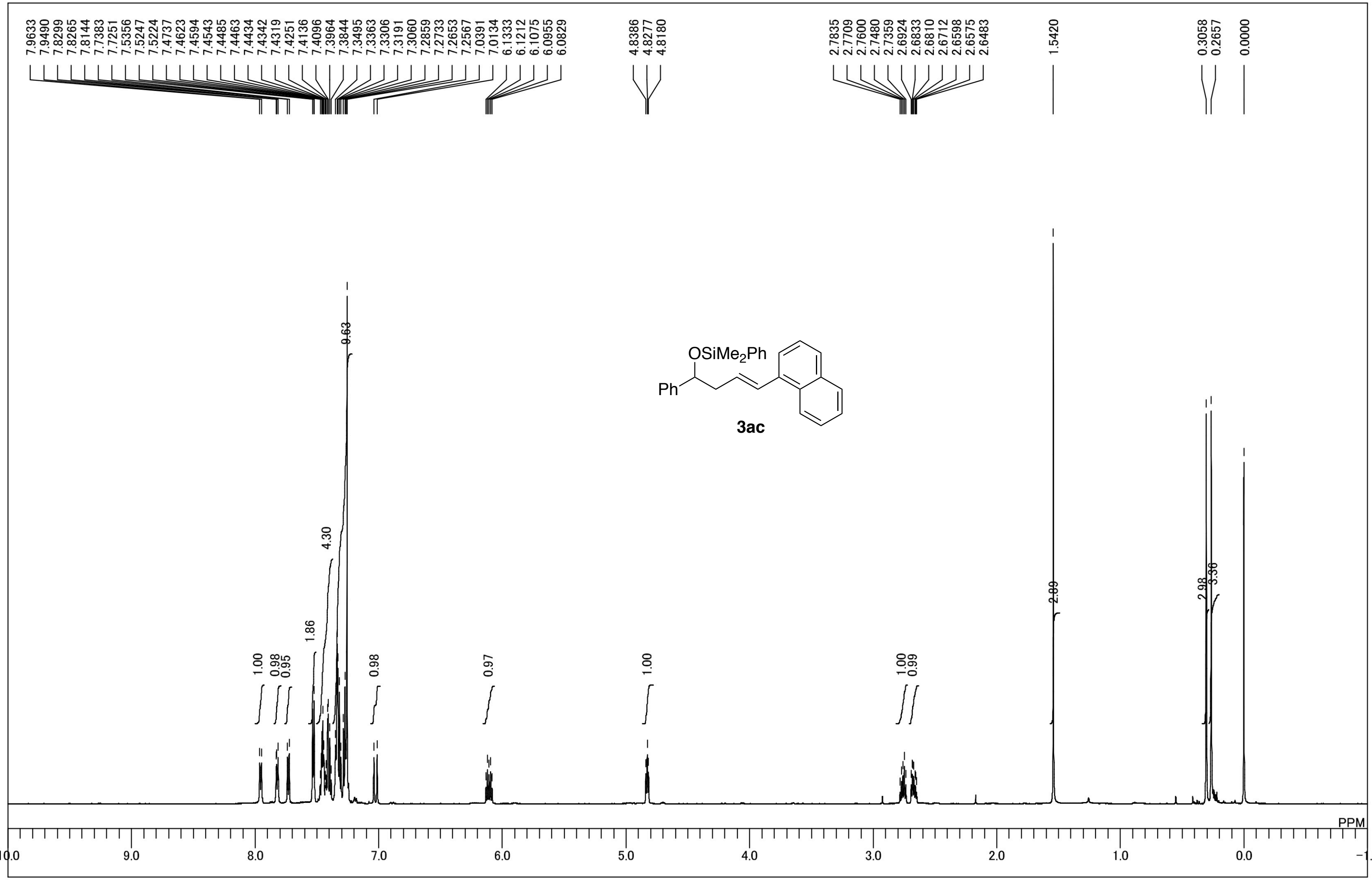

^{1}H NMR spectrum of **3ac**

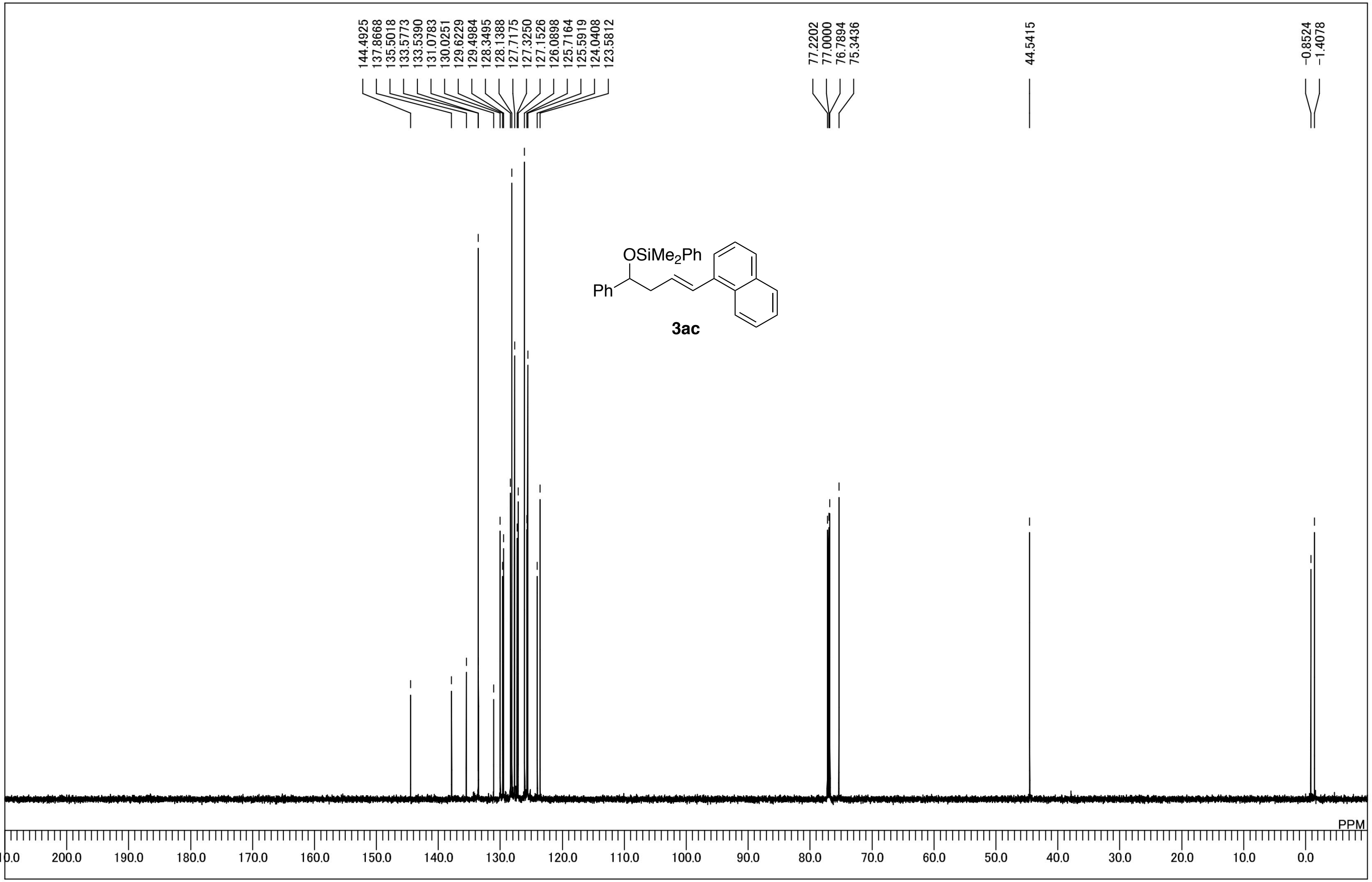

^{13}C NMR spectrum of **3ac**

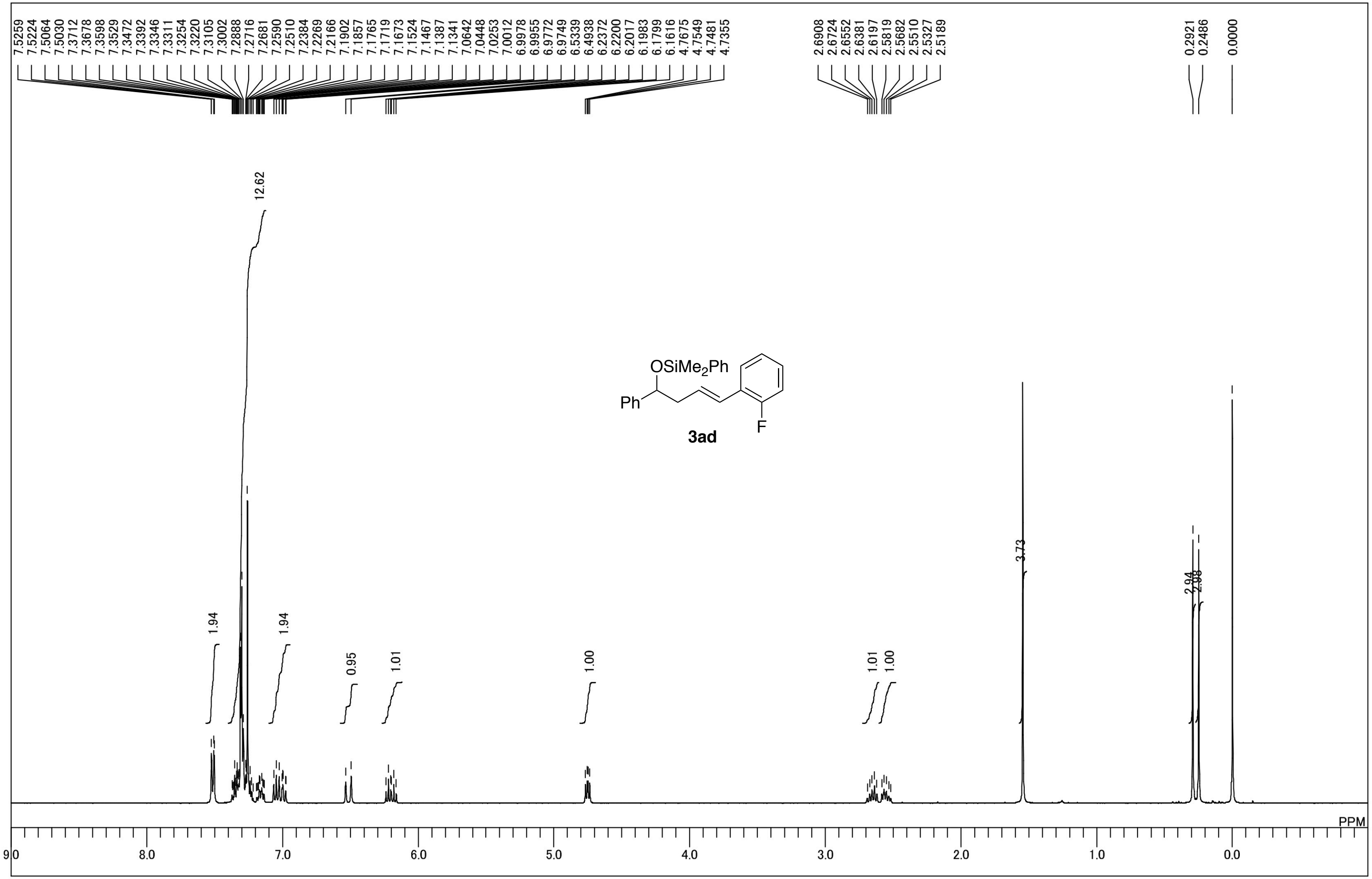

^{1}H NMR spectrum of **3ad**

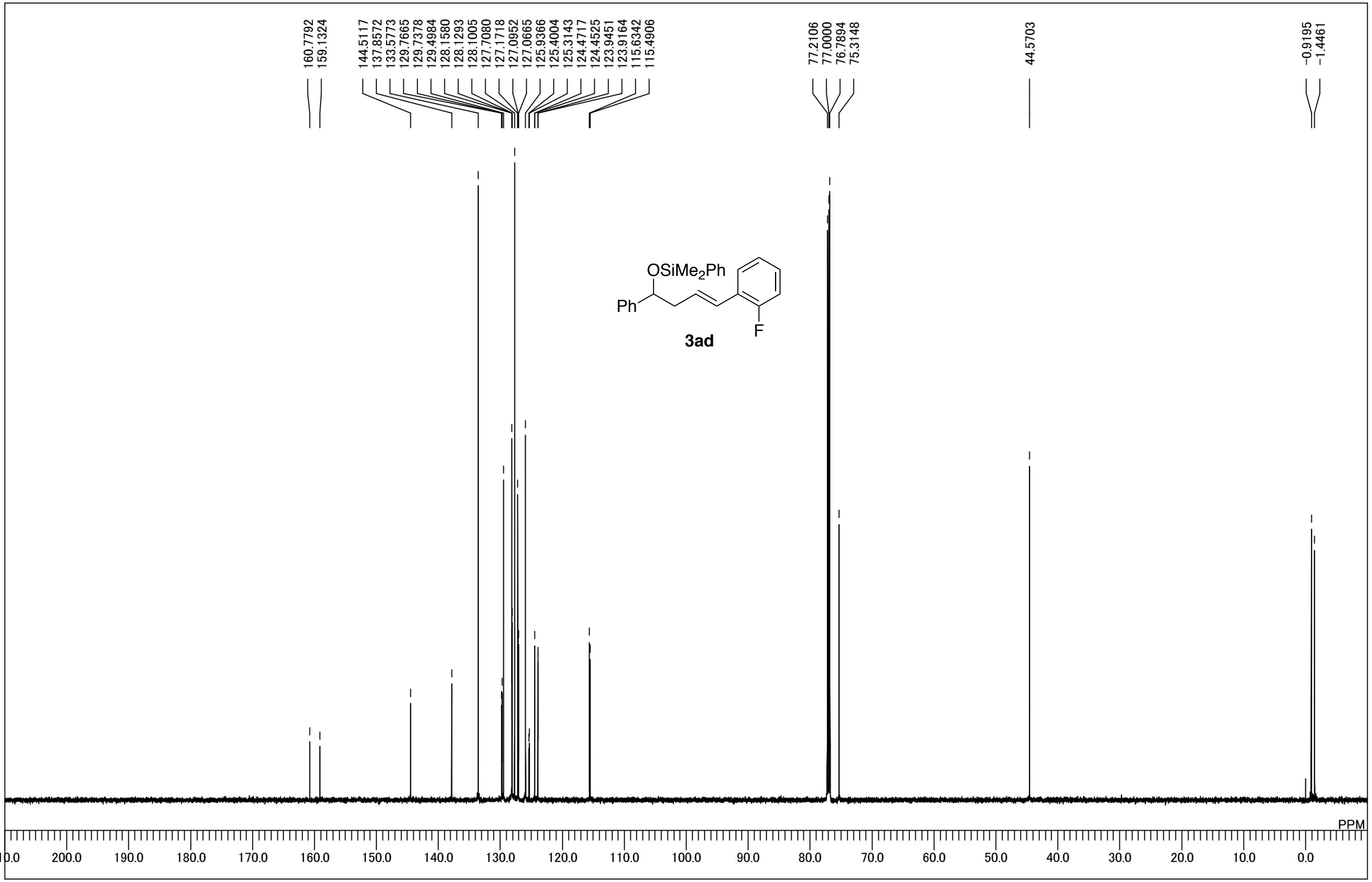

^{13}C NMR spectrum of **3ad**

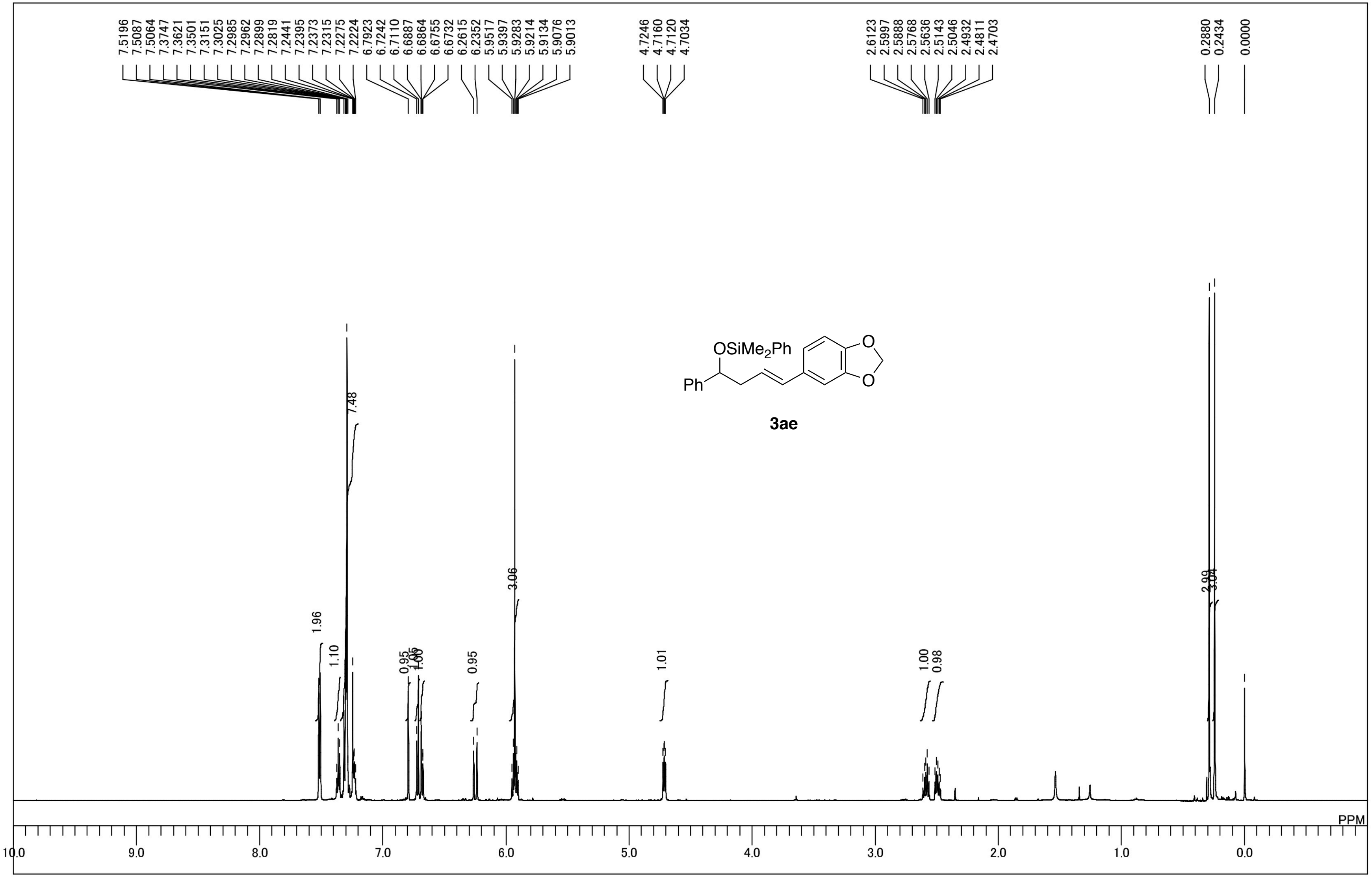

^{1}H NMR spectrum of **3ae**

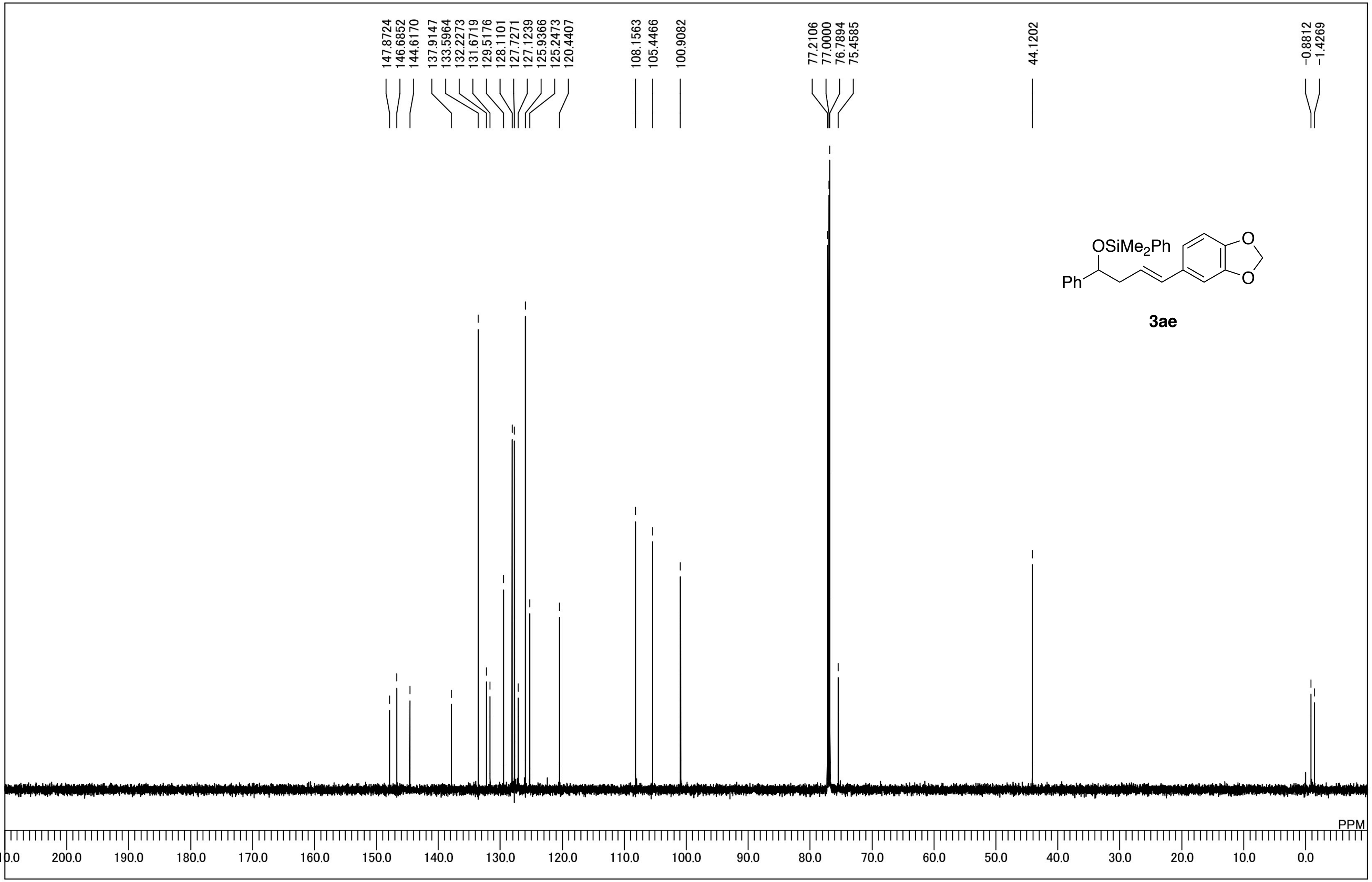

^{13}C NMR spectrum of **3ae**

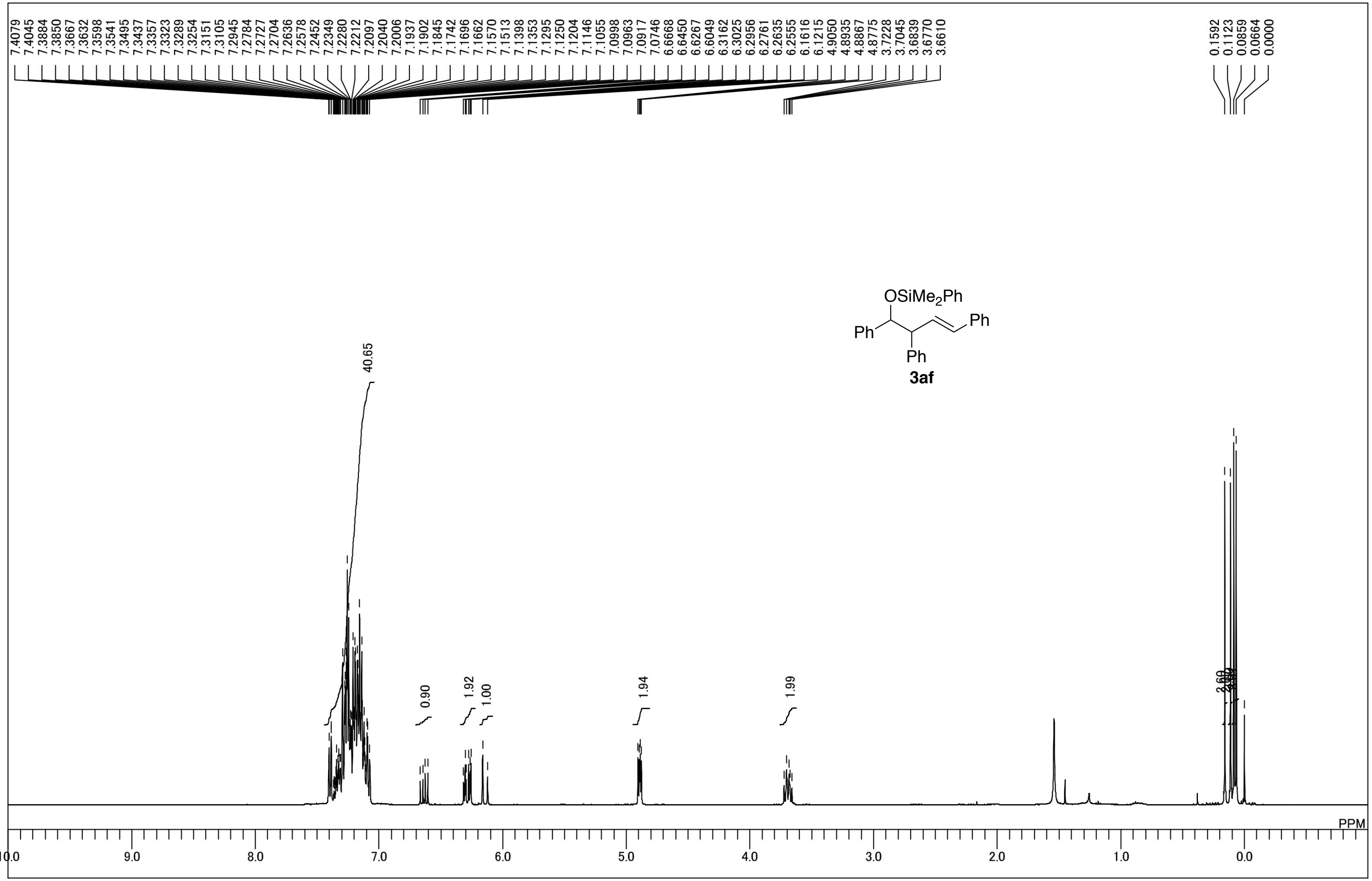

^{1}H NMR spectrum of **3af**

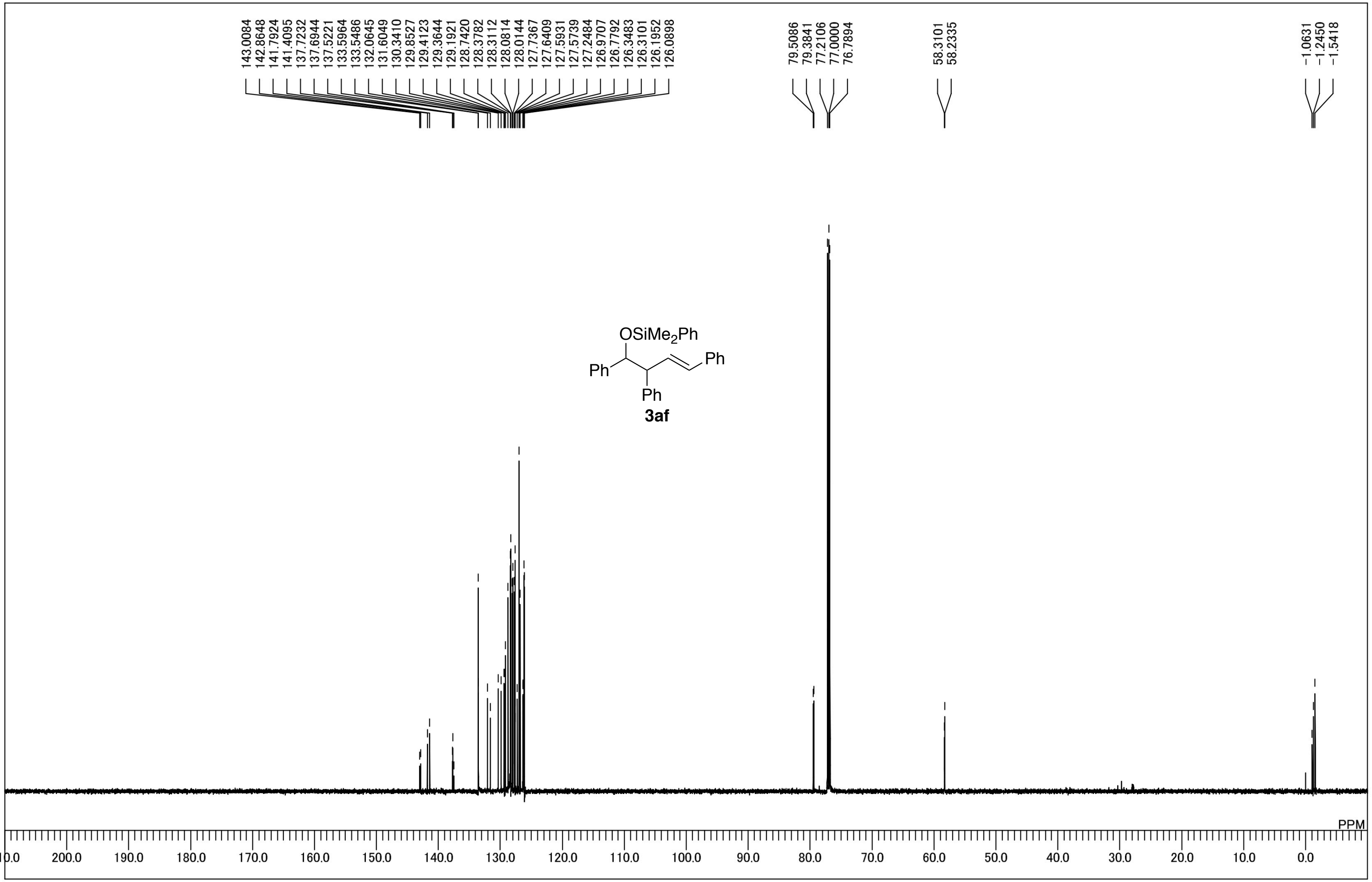

^{13}C NMR spectrum of **3af**
